# Giant Cell Granuloma: Two Expressions in Pediatric Population

**DOI:** 10.5005/jp-journals-10005-1482

**Published:** 2017-02-01

**Authors:** Chitrita G Mukherjee, Uday Mukherjee, Anju Bansal, Madhushree Mukhopadhyay

**Affiliations:** 1 Professor and Head, Department of Pedodontics and Preventive Dentistry Buddha Institute of Dental Sciences & Hospital, Patna, Bihar India; 2 Ex-Professor, Department of Oral and Maxillofacial Surgery, Buddha Institute of Dental Sciences & Hospital, Patna, Bihar, India; 3 Reader, Department of Pedodontics and Preventive Dentistry Buddha Institute of Dental Sciences & Hospital, Patna, Bihar India; 4 Postgraduate Student, Department of Pedodontics and Preventive Dentistry Buddha Institute of Dental Sciences & Hospital, Patna, Bihar India

**Keywords:** Central, Children, Giant cell granuloma, Peripheral.

## Abstract

A granuloma is a collection of epithelioid histiocytes that is often associated with multinucleated giant cells, and is considered widely to be a non-neoplastic lesion, although some lesions demonstrate aggressive behavior similar to that of a neoplasm. The diagnosis of giant cell granulomas (central and peripheral) is confirmed by histopathologic examination. Early detection and excision are important to minimize potential dentoalveolar complications. The following article consists of case reports of central and peripheral giant cell granuloma (PGCG), and discussion about the diagnosis and management of such lesions.

**How to cite this article:** Mukherjee CG, Mukherjee U, Bansal A, Mukhopadhyay M. Giant Cell Granuloma: Two Expressions in Pediatric Population. Int J Clin Pediatr Dent 2018;11(1):46-49.

## INTRODUCTION

Solitary gingival enlargements, which usually occur as a reactive response to a local irritation, are a relatively common finding in children. In some cases, these undergo progressive growth and reach a considerable size, which compromises oral function.^1^ An example of such enlargement is a giant cell granuloma of the jaws, which can be of two types:

1. Peripheral exophytic lesion occurring on the gingival/ alveolar crest.
2. Centrally located lesion within the jaw, facial bones, or skull.

These are benign reactive lesions, which are of unknown etiology and pathogenesis.^2^ Both lesions are characterized by the presence of several multinucleated giant cells and mononuclear stromal cells in a fibrous connective tissue matrix.^3^ The most common treatment is surgical, which may range from curettage to *en bloc* resection.^4^ This article reports two cases of giant cell granuloma, central and peripheral, and describes the presentation, investigation, diagnosis, and treatment.

## CASE REPORTS

### Case 1

**Fig. 1:**
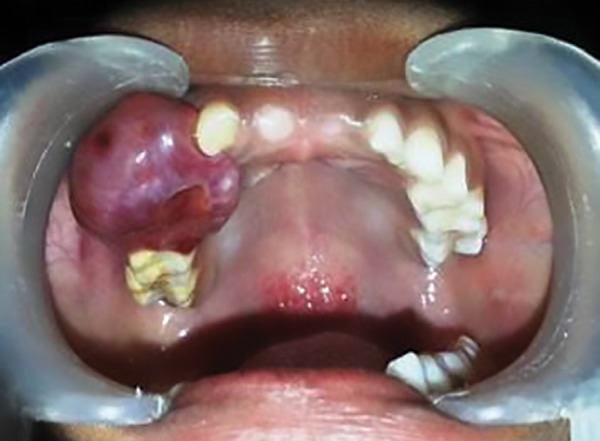
Intraoral view of lesion

An 8-year-old boy reported with a swelling in the right upper front region of the jaw for the past 6 months. History revealed that initially, the swelling was small in size, and increased gradually, and did not respond to antibiotics prescribed by the physician. Intraoral clinical examination revealed a single, sessile, firm, localized, nontender swelling in the edentulous region, extending from distal margin of 52 till the mesial margin of 55 (approximately 3 cm × 4 cm). The mucosa over the swelling was erythematous with a purple hue, with indentations on the surface due to contact with teeth of the opposing arch (Fig. 1). Calculus deposits were observed on the adjacent teeth.

**Fig. 2:**
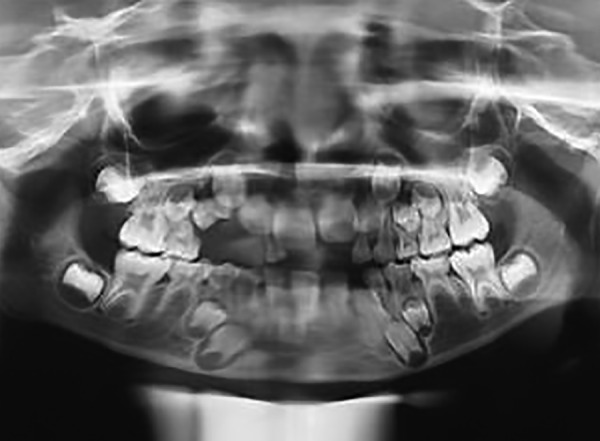
Orthopantomogram

Orthopantomogram revealed a poorly circumscribed radiolucency extending from region of 52 to 55 (Fig. 2).

**Fig. 3:**
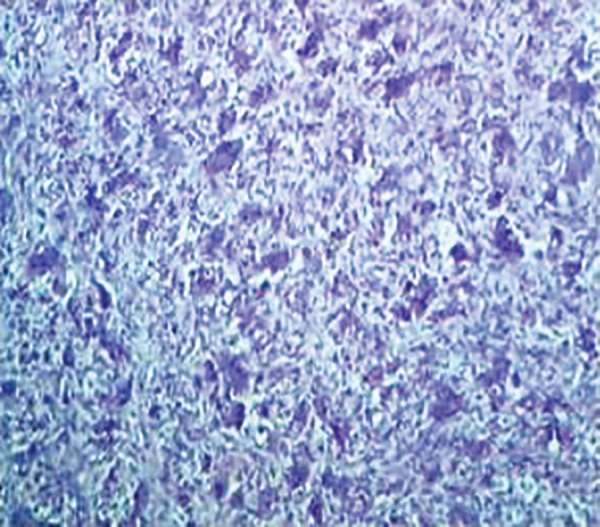
Photomicrograph showing multiple multinucleated giant cells in collagenous stroma (hematoxylin and eosin stain, 10* magnification)

**Fig. 4:**
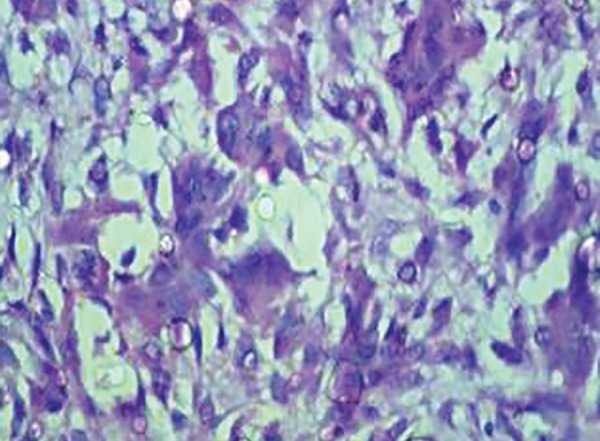
Photomicrograph showing large multinucleated giant cells in stromal tissue (hematoxylin and eosin stain, 40* magnifications)

Written consent from the parents was obtained, followed by incisional biopsy. The histopathological sections stained with hematoxylin and eosin revealed superficial ulcerated stratified squamous epithelium. The lamina propria revealed profuse multinucleated giant cells in highly cellular and vascular connective tissue stroma. The stromal tissue also showed extravasated red blood cells and hemosiderin pigment (Figs 3 and 4).

**Figs 5A and B:**
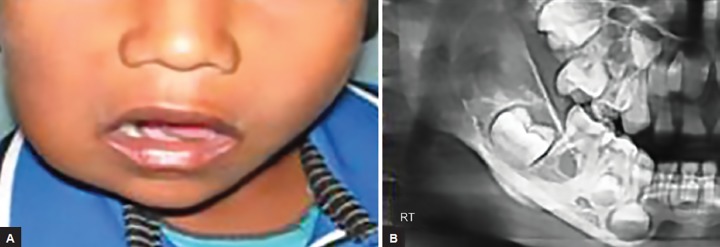
Preoperative photograph and orthopantomogram

Thus, the diagnosis of PGCG was confirmed. Excision of the entire growth was carried out after a week, under local anesthesia, followed by placement of sutures.

### Case 2

A 4-year-old girl reported with slowly enlarging bony hard swelling in the lower right back region of face, causing gross facial deformity. Orthopantomogram revealed multilocular radiolucent shadow extending from mandibular right first deciduous molar till anterior border of ramus of the mandible (Fig. 5).

**Fig. 6:**
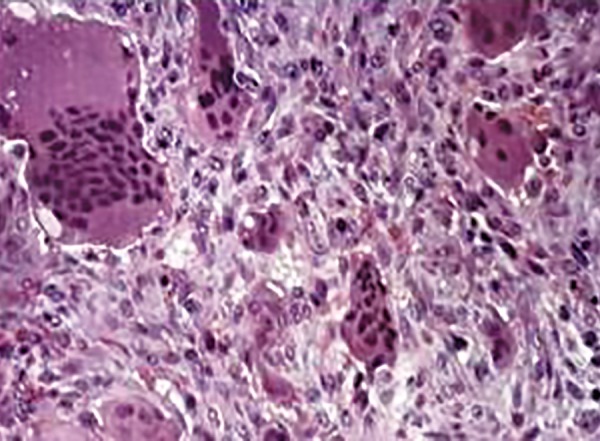
Photomicrograph showing osteoclast type giant cells alternate with fibrovascular stroma (10* magnification)

Written consent from the parents was obtained, followed by incisional biopsy. Photomicrograph of the section stained with hematoxylin and eosin revealed multiple multinucleated giant cells in highly cellular and vascular connective tissue stroma. The stromal tissue showed bony trabeculae, osteoids, and hemosiderin pigments. The case was finally diagnosed as central giant cell granuloma (CGCG) (Fig. 6).

Surgical management involved meticulous removal of the lesion intraorally along with additional curettage.

**Fig. 7:**
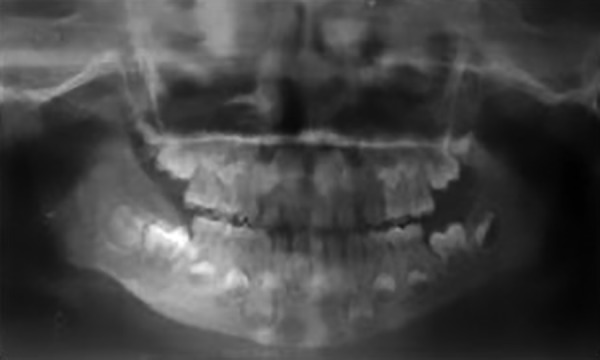
Postoperative orthopantomogram

Postoperative panoramic radiograph showed evidence of satisfactory healing (Fig. 7).

## DISCUSSION

The PGCG of the oral cavity is more commonly seen in the mandibular arch,^5^ clinically manifesting as a firm sessile or pedunculated nodule.^6^ It arises usually in response to local irritation, mainly from the gingival connective tissue, periodontal membrane, or periosteum of the alveolar ridge.^7^

Radiographic features are usually nonspecific. Sometimes, the crest of the interdental bone or the alveolar margin exhibits signs of superficial destruction when the granuloma occurs associated with the teeth. Superficial erosion with peripheral “cuffing” of the underlying bone is seen in cases where the granuloma arises from the edentulous ridge.^8^

The differential diagnosis of PGCG includes pyo-genic granuloma, fibrous epulis, inflammatory fibrous hyperplasia, peripheral ossifying fibroma, peripheral odontogenic fibroma, and papilloma. Histopathological examination confirms the diagnosis.^9^ It is characterized by a nonencapsulated mass of tissue, which consists of a reticular and fibrillar connective tissue stroma with plump, ovoid, and fusiform fibroblasts and multinucleated giant cells.^10^ Management includes eliminating the entire base of the growth along with any local irritating factors.

In children, reactive oral lesions like PGCG may exhibit a rapid growth rate and, within several months of initial diagnosis, reach significant size. These soft tissue nodules may sometimes be quite aggressive and cause bone resorption, interfere with eruption of teeth and its movement of varying degrees. Radiographs aid in confirming that PGCG does not represent a central bone lesion with cortical perforation along with soft tissue extension, but from the oral mucosa. Early detection results in decreased risk of tooth and bone loss.^1^

Central giant cell granuloma, a relatively uncommon benign bony lesion, exhibits a variably aggressive nature, occurring predominantly in children and young adults. It is usually asymptomatic, discovered during routine radiographic examinations, or when the painless bony expansion is noted by the patient or the parents.^10^

Serum parathyroid hormone levels should be analyzed in such cases, in order to rule out the diagnosis of Brown’s tumor, as seen in cases of hyperparathyroidism.^10^

Some reports have divided the lesions of CGCG into two categories:

1. Nonaggressive lesions with a slow growth rate, without cortical perforation, and may show new bone formation;
2. Aggressive lesions grow quickly and are associated with pain, cortical perforation, and root resorption. Paresthesia of the lip is an occasional finding.^10^

Establishment of a definitive diagnosis relies on a combination of the clinical and radiological findings with biochemistry and histopathology. Conventional management is by curettage or resection, which may result in loss of teeth, or developing tooth germs. Non-surgical treatment includes systemic calcitonin therapy and intralesional injections with corticosteroids, which may be considered in case of children, since it does not involve immediate loss of teeth or bone structure but may require eventual future surgical treatment.^11^

## CONCLUSION

The choice of treatment is influenced by clinical behavior of the lesion. The patient’s age, site, and extension of the growth are also contributing factors. Early detection in children enables conservative management, resulting in decreased undesirable loss of oral structures.
